# Response to the 2009-H1N1 influenza pandemic in the Mekong Basin: surveys of country health leaders

**DOI:** 10.1186/1756-0500-4-361

**Published:** 2011-09-16

**Authors:** Melinda Moore, David J Dausey

**Affiliations:** 1 Health Unit, RAND Corporation, Arlington VA, USA; 2 Mercyhurst Institute for Public Health, Mercyhurst College, Erie PA, USA

## Abstract

**Background:**

Soon after the 2009-H1N1 virus emerged as the first influenza pandemic in 41 years, countries had an early opportunity to test their preparedness plans, protocols and procedures, including their cooperation with other countries in responding to the global pandemic threat. The Mekong Basin Disease Surveillance cooperation (MBDS) comprises six countries - Cambodia, China (Yunnan and Guangxi Provinces), Lao People's Democratic Republic, Myanmar, Thailand and Vietnam - that formally organized themselves in 2001 to cooperate in disease surveillance and control. The pandemic presented an opportunity to assess their responses in light of their individual and joint planning. We conducted two surveys of the MBDS leadership from each country, early during the pandemic and shortly after it ended.

**Results:**

On average, participants rated their country's pandemic response performance as good in both 2009 and 2010. Post-pandemic (2010), perceived performance quality was best for facility-based interventions (overall mean of 4.2 on a scale from 1 = poor to 5 = excellent), followed by surveillance and information sharing (4.1), risk communications (3.9) and disease prevention and control in communities (3.7). Performance was consistently rated as good or excellent for use of hotlines for case reporting (2010 mean of 4.4) and of selected facility-based interventions (each with a 2010 mean of 4.4): using hospital admission criteria, preparing or using isolation areas, using PPE for healthcare workers and using antiviral drugs for treatment. In at least half the countries, the post-pandemic ratings were lower than initial 2009 assessments for performance related to surveillance, facility-based interventions and risk communications.

**Conclusions:**

MBDS health leaders perceived their pandemic responses effective in areas previously considered problematic. Most felt that MBDS cooperation helped drive and thus added value to their efforts. Surveillance capacity within countries and surveillance information sharing across countries, longstanding MBDS focus areas, were cited as particular strengths. Several areas needing further improvement are already core strategies in the 2011-2016 MBDS Action Plan. Self-organized sub-regional cooperation in disease surveillance is increasingly recognized as an important new element in global disease prevention and control. Our findings suggest that more research is needed to understand the characteristics of networking that will result in the best shared outcomes.

## Background

As the world prepared for a pandemic because of the emergence and spread of avian influenza A/H5N1 in the Eastern Hemisphere beginning in late 2003, a different pandemic influenza strain (2009-H1N1) emerged in the Western Hemisphere in 2009 [1]. The first report of influenza cases caused by a novel influenza A/H1N1 virus was from the United States on April 17, 2009, describing two cases from California [2]; cases subsequently confirmed as due to the same virus had appeared in Mexico beginning in mid-March [3]. Beginning on April 24, 2009, the World Health Organization (WHO) issued nearly daily, occasionally twice daily, "situation updates" on the spread of the virus, now known as 2009-H1N1 [4]. On June 11, the WHO situation update #47 indicated 28,774 confirmed cases in 74 countries [5]; on that date, the WHO Director-General Dr. Margaret Chan declared that disease spread had reached pandemic status [6]. The pandemic continued into 2010. On August 10, 2010, Dr. Chan announced transition to the post-pandemic period, signalling the end of the pandemic [7].

The 2009-H1N1 virus that emerged as the first influenza pandemic in 41 years presented a less severe clinical picture compared to the worst-case scenario for which planners around the world had been preparing themselves [8]. By late spring 2009, numerous countries were experiencing the "first wave" of disease due to the novel H1N1 virus, the Southern Hemisphere was about to enter its winter influenza season, and the Northern Hemisphere was concerned about a "second pandemic wave" in its own upcoming fall-winter influenza season. Thus, during spring 2009 countries had the opportunity to assess their pandemic preparedness against the novel influenza H1N1 virus, in preparation for expected upcoming surges in disease [9,10].

The Mekong Basin Disease Surveillance cooperation (MBDS) comprises six countries - Cambodia, China (originally Yunnan and, since 2008, also Guangxi Province), Lao People's Democratic Republic, Myanmar, Thailand and Vietnam - that formally organized themselves in 2001 to collaborate in sub-regional infectious disease surveillance and control [11]. Soon after the first Memorandum of Understanding was signed by the countries' health ministers in 2001, programming focused on building epidemiology capacity, then evolved in 2003 to establish a handful of "cross-border sites" where cooperative surveillance, outbreak investigation by Rapid Response Teams and related disease control activities were organized [12].

In 2006-2007, MBDS leaders expanded their programming to include pandemic influenza preparedness, through a series of typically multi-sector national and provincial level tabletop exercises in each country which culminated in a broadly multi-sector regional tabletop exercise in early 2007 [13]. According to the United Nations System Influenza Coordinator's office, this was the world's first multi-sector, regional pandemic influenza exercise [14]. At the time of those exercises, the world's focus was on influenza H5N1, and many experts expected that a pandemic would arise from Asia more broadly and in Southeast Asia in particular [15,16].

Countries in Southeast Asia face a number of important challenges to mounting effective responses to emerging infectious diseases, including pandemics. Resource-poor environments are associated with limited infrastructures -- both the traditional limitations in rural areas and infrastructures that cannot keep pace with the newer realities of urbanization, including rapid population growth and inadequate sanitation systems [17]. Such countries are typically substantially dependent on external support to help build their public health capacity and pandemic preparedness [18,19]. Their pandemic planning must take into account limited expectations regarding access to pharmaceutical interventions during a pandemic, such as vaccines and antiviral agents [20]. By 2006, only some countries in Asia had engaged in any significant planning for pandemic influenza [21]. Thus, pandemic preparedness planning at that time was particularly compelling for MBDS, located in the epicenter of expected pandemic emergence and coupled with these additional challenges.

There is a growing recognition in the global health community that routine disease surveillance, prevention and control, and especially preparing for pandemics and other large-scale public health emergencies require regional and sub-regional planning and cooperation that supplements and augments purely national efforts [22,23]. Formally established regional and sub-regional networks driven by users can help to facilitate this process. For example, these networks have helped to facilitate the implementation of the International Health Regulations [24-26]. Despite this increased recognition, little has been written about these regional and sub-regional networks in the literature [27]. The response to 2009-H1N1 involved activating and using many of these networks, yet we know very little about how these networks function or whether or not they led to improved outcomes.

In an effort to better understand how national and sub-regional pandemic influenza preparedness planning helped in the response to 2009-H1N1 in Southeast Asia, we surveyed MBDS leaders early during the pandemic and again shortly after it ended. The purpose was to assess countries' perceptions regarding the effectiveness of their responses including both initial and overall responses. These surveys were carried out for practical purposes for the six MBDS member countries, to aid their ongoing pandemic response and then reflect on further improvements needed to strengthen their respective public health systems and their cooperation. The surveys thus provide lessons for these six countries, but also information that may be valuable to other countries and other sub-regional groups of countries cooperating in disease surveillance and control.

## Methods

### Data collection

We conducted two surveys of the MBDS leadership. The surveys were in English, the longstanding official language of communication within MBDS. One MBDS leader for each country, known as the "Country Coordinator", completed each survey. These leaders are fully conversant in English. The first survey was distributed in person during an MBDS meeting in June 2009; some were completed and returned on site, and the remainder were returned by e-mail. The second survey was e-mailed to the MBDS Country Coordinators through the MBDS Coordination Office, completed electronically and then returned by e-mail. The first survey was completed between June 26 and July 10, 2009, within one month of the official WHO declaration of the pandemic on June 11; the second survey was completed between December 7, 2010 and January 16, 2011, approximately four months after official WHO declaration of the pandemic's end. Information to complement the information collected in the surveys was obtained from WHO situation updates [4]. Because the surveys did not involve any sensitive questions about individuals and were purely informational in nature, they were exempted from Institutional Review Board review. Participants gave verbal informed consent to participate in the research.

### Survey structure and content

The surveys contained structured and semi-structured questions and were designed to be self-administered. The first survey and the second survey contained the same questions with the exceptions noted below. Both structured and semi-structured questions concentrated on three broad areas previously designated as high priorities by MBDS countries from the series of tabletop exercises in 2006-2007: surveillance and information sharing, disease prevention and control in health facilities and the community, and risk communications.

Participants were first asked to indicate the number of confirmed cases of 2009-H1N1 their country had experienced at the time they completed the first survey. Additional structured questions required survey participants to indicate whether or not their country undertook 32 separate activities divided across the three broad areas. These activities were identified as needed response capabilities by MBDS leaders in their regional pandemic influenza tabletop exercise [13]. In the first survey only, participants were asked when they first undertook each action during the evolution of the pandemic: when cases were in the Americas, when cases were in their region, when cases were in their country, after the pandemic had been declared, or not at all. In both surveys, participants were asked to rate on a 5-point Likert Scale (where 1 = very poor and 5 = excellent) their subjective opinion as to the effectiveness of each activity undertaken in their response to the pandemic. Using the same scale they were also asked to rate how effective the same action would have been, hypothetically, in 2006 - before the MBDS exercises and extensive regional pandemic response planning. Finally, participants were asked to use a 5-point Likert Scale (where 1 = not at all and 5 = very much) to rate how much eleven planning-related factors drove or contributed to the success of their pandemic responses.

Semi-structured questions asked participants to provide narrative responses relating to the three broad activity areas discussed above, plus a fourth area - coordination within MBDS: "What went right and why?" and "What needs improvement and what will you plan to do to make these improvements?" Participants were also able to provide narrative comments on improvements needed in connection with any of the 32 specific activities, to complement their Likert scale ratings.

### Data analysis and synthesis

Responses to structured questions from both surveys were compiled and analyzed using Microsoft Excel 2007. Assessment of when countries initiated specific activities was based on counting the number of countries with early initiation (value of 1 or 2) and later initiation responses (value of 3 or 4) and then calculating the percentage of all responses that were initiated early. Countries that did not undertake the activity were excluded from the calculations. Mean values of Likert scale responses were calculated separately for each survey for each of the 32 specific activities across countries and each country within each of the three broad areas. Overall means for each of the three broad areas were calculated for both survey periods. All calculations included only affirmative responses; items not reported or reported as an activity not undertaken were excluded from both numerator and denominator for each calculation. Comparisons between perceived effectiveness of actual performance (in 2009 and again in 2010) and hypothetical performance (had each activity been undertaken in 2006) were calculated as the difference in values for actual and hypothetical performance for each activity for each survey (2009, 2010).

Responses to semi-structured questions were analyzed using recursive abstraction. Country level responses to each question were examined to identify common themes. The limited amount of data allowed for comparisons of verbatim responses. Themes that appeared in the responses of more than one country were selected for additional analysis. Qualitative summaries of these themes were developed.

## Results

### Initial spread of 2009-H1N1 in the region

At the time the Country Coordinators completed the first survey, all countries had reported at least one confirmed case of 2009-H1N1 (Table 1). Thailand was the first MBDS country to report confirmed H1N1 cases, on May 13, 2009; at that time, 33 countries had reported cases to the WHO. Thailand and Vietnam both reported their first cases before June 11, when the pandemic was declared; the first cases in the four other MBDS countries were reported after 2009-H1N1 had reached pandemic status, with Myanmar as the last country to report its first case on July 1. By that date, Thailand had already reported 1414 cases and Vietnam, 123 cases.

**Table 1:** Confirmed influenza H1N1 cases reported by MBDS countries to WHO and global situation, June-July 2009^1^

Country	Country Reports			WHO Reports		
				
				WHO situation update on date country's first cases reported^1^:		
	
	Date Survey Completed	Date of first case/s in WHO report	Total # cases in WHO July 1 report	Situation update #	# of countries	# of cases worldwide
Thailand	June 26	May 13	1414	27	33	5728

Vietnam	July 6	June 1	123	42	62	17,410

China (Yunnan)^2^	June 26	June 15	NA	49	76	35,928

China (Guangxi)^2^	June 26	NA	NA	NA	NA	NA

Laos	June 26	June 19	3	51	88	44,287

Cambodia	June 26	June 24	6	53	102	55,867

Myanmar	July 10	July 1	1	56	112	77,201

1. WHO data come from [4]

2. WHO situation updates did not specifically address cases in China by province; an online source reported the first case from Yunnan on June 15, but similar information was not available for Guangxi Province; NA = not applicable/not available

### When did countries initiate specific response activities?

Table 2 presents details of whether or not countries had undertaken each of the 32 specific activities at the time of the June 2009 survey and, if so, when during the evolution of the pandemic they initiated the activity. Nearly all countries established a case definition for 2009-H1N1 very early, when cases first appeared in the Americas, and most countries also instituted active case finding at hospitals and clinics and established a hotline for reporting cases during the early stages of the evolving pandemic. Laos and both Yunnan and Guangxi Provinces in China each reported initiating most of their activities quite early in the evolution of the pandemic, when cases first appeared in the Americas or shortly thereafter; the other MBDS countries mostly initiated activities when 2009-H1N1 had arrived in their region or country. All countries mobilized their Rapid Response Teams for epidemiologic investigation and response, though at different times in the evolution of the pandemic. The occurrence and timing of communications with established MBDS cross-border sites, with other MBDS countries, and with the MBDS Coordinator varied across the six countries.

**Table 2:** Time at which countries first implemented specific activities^1,2, 3^

ACTIVITY	CAMB	CYUN	CGUA	LAOS	MYAN	THAI	VIET	% 1 or 2
***Surveillance and Information Sharing***								

Development/use of H1N1 case definition	2	1	1	1	1	1	2	100

Active case finding - hospitals/clinics	4	1	1	2	1	2	3	86

Active case finding - specific communities	3	3	3	2	1	2	3	43

Active case finding - ports of entry	3	1	1	1	3	1	3	57

Electronic reporting of cases	1	1	1	0	3	2	3	57

Hotline for reporting possible cases	2	1	1	1	1	3	3	71

Initial testing in district or provincial lab	0	1	1	0	0	2	3	43

Rapid Response Team mobilization	3	1	1	1	3	NR	3	50

Communication with cross-border site(s)	1	1	0	1	3	NR	2	67

Sharing information with MBDS country	1	0	0	1	3	NR	2	50

Sharing information with MBDS Coordinator	1	3	0	1	3	NR	2	50

***Disease Prevention and Control - Health Care Facilities***								

Communications with healthcare workers	1	1	3	2	1	NR	3	67

Use of specific hospital admission criteria	1	1	0	2	1	NR	3	67

Preparation or use of isolation areas	1	1	1	2	1	NR	3	83

Use of PPE for healthcare workers	1	1	1	3	1	NR	3	67

Use of antiviral drugs for treatment	3	1	3	3	2	NR	3	33

Use of antiviral drugs for prophylaxis	3	2	3	3	3	NR	3	17

More attention to hospital infection control	1	1	1	3	4	NR	3	50

(More) preparation for medical surge	1	1	3	3	4	NR	3	33

***Disease Prevention and Control - Communities***								

Containment measures (e.g., from WHO)	3	2	1	1	4	NR	NR	50

Border control measures	1	0	1	2	1	NR	NR	80

Restrictions of travel	4	0	0	0	0	0	NR	0

Restrictions of public gatherings	4	0	0	0	0	0	NR	0

Closure of any schools	4	0	0	0	4	NR	NR	0

***Risk Communications***								

Spokesperson(s) identified	1	2	3	1	1	NR	3	50

Public risk communications - targeted	1	2	3	2	3	NR	3	50

Public risk communications - everywhere	3	2	3	3	1	NR	3	33

Consistency in messages for the public	1	1	1	3	1	NR	3	67

Messages aimed to avoid public panic	1	1	1	3	1	NR	3	67

Communications with any MBDS country	1	0	0	1	3	NR	2	50

Communications with MBDS Coordinator	3	0	0	1	3	0	2	29

Communications with any external partner	3	0	0	1	1	0	2	43

1. CAMB = Cambodia; CYUN = China Yunnan-Province; CGUA = China Guangxi Province; LAOS = Laos; MYAN = Myanmar; THAI = Thailand; VIET = Vietnam.

2. 0 = Not implemented; 1 = when cases were in the Americas; 2 = when cases were in the Asia region; 3 = when cases were in their country; 4 = after pandemic was declared.

3. NR = No response

Cambodia, China and Myanmar each reported that they began preparing their respective health care systems for 2009-H1N1 when cases first appeared in the Americas, e.g., through communication with health workers, use of specific hospital admission criteria, preparation of isolation areas and of personal protective equipment for health workers, increased attention to hospital infection control, and preparation for medical surge. Laos began such activities mostly when cases were in the region or country, and Vietnam, when cases had arrived in the country. An increased focus on PPE for health care workers and preparations for hospital isolation and care were the earliest activities undertaken to prepare the health care system in most MBDS countries, at whatever stage such preparations were initiated.

Cambodia, China's Guangxi Province, and Myanmar each reported that they began border control measures very early, when known cases were limited to the Americas. Cambodia, both Chinese provinces, Laos and Myanmar each reported instituting containment measures, but at different points in the evolution of the pandemic. Cambodia restricted travel (the only MBDS country to do so) and public gatherings and closed schools after the pandemic had been declared. Myanmar was the only other country to implement any such restrictions, also closing schools once the pandemic was announced.

Cambodia, Laos and Myanmar each reported that they identified communications spokespersons early in the evolution of the pandemic. Message consistency and communications to avoid public panic were early priorities for risk communications in Cambodia, China and Myanmar. Communications within the MBDS community - with other countries and with the MBDS Coordinator in Bangkok - was an early priority for some countries, but was undertaken later or not at all by others.

### How did countries perceive their response performance in 2009 and 2010?

Table 3 presents each country's subjective ratings of its own performance early during the pandemic and shortly post-pandemic. Some activities that had not been implemented at the time of the first survey in June 2009 were subsequently undertaken and were rated on the second survey. On average, participants rated their country's performance both early and post-pandemic as good. Comparison of Tables 2 and 3 suggests that performance was judged to be better for activities initiated earlier during the evolution of the pandemic with regard to surveillance and information sharing in China-Yunnan, Laos, Myanmar and Thailand, and to a lesser extent, risk communications in Cambodia and Myanmar. Post-pandemic, the broad area that received the highest overall mean rating was disease prevention and control in health care facilities (4.2), followed by surveillance and information sharing (4.1), risk communications (3.9) and disease prevention and control in communities (3.7). More countries voluntarily chose to not implement community-based interventions, compared to activities in the other broad areas. Countries consistently rated their performance as good or excellent for use of hotlines for case reporting (2010 mean of 4.4) and of selected facility-based interventions (each with a 2010 mean of 4.4): using hospital admission criteria, preparing or using isolation areas, using PPE for healthcare workers and using antiviral drugs for treatment.

**Table 3:** Subjective ratings of actual response performance^1, 2, 3^

ACTIVITY	CAMB		CYUN		CGUA		LAOS		MYAN		THAI		VIET		MEAN	
	
	'09	'10	'09	'10	'09	'10	'09	'10	'09	'10	'09	'10	'09	'10	'09	'10
***Surveillance and Information Sharing***																

Development/use of case definition	5	5	4	4	4	4	5	4	NR	5	4	4	5	4	4.5	4.3

Active case finding/hospitals & clinics	5	4	5	4	5	4	3	4	NI	5	3	4	5	4	4.3	4.1

Active case finding/communities	5	4	3	4	4	4	3	4	NR	5	3	4	3	4	3.5	4.1

Active case finding/ports of entry	5	4	4	4	5	4	5	NI	NR	5	4	4	4	4	4.5	4.2

Electronic reporting of cases	5	4	5	5	5	4	NI	3	NR	4	4	4	3	4	4.4	4.0

Hotline for reporting possible cases	5	5	4	5	3	4	3	4	5	5	3	4	4	4	3.9	4.4

Initial testing in district/provincial lab	NI	4	5	4	4	4	NI	NI	NI	4	3	NI	2	4	3.8	4.0

Any other active surveillance activities	4	5	5	3	4	4	4	3	4	4	3	2	5	4	4.1	3.6

Rapid Response Team mobilization	5	5	5	4	5	4	NR	4	4	4	3	5	5	4	4.5	4.3

Communication w/cross-border site(s)	5	4	5	3	NI	4	NR	4	NR	4	3	NI	3	4	4.0	3.8

Sharing info w/MBDS country	5	5	NI	3	NI	4	NR	4	NR	4	3	NI	4	4	4.0	4.0

Sharing info w/MBDS Coordinator	5	5	4	4	NI	4	NR	4	NR	4	3	NI	4	4	4.0	4.2

**MEAN**	4.9	4.5	4.5	3.9	4.3	4.0	3.8	3.8	4.3	4.4	3.3	3.9	3.9	4.0	**4.1**	**4.1**

***Disease Prevention and Control - Health Care Facilities***																

Communications w/health workers	4	4	5	4	4	4	NR	4	NR	5	3	4	4	4	4.0	4.1

Use hospital admission criteria	5	5	5	4	NI	4	NR	4	NR	5	4	5	4	4	4.5	4.4

Preparation or use of isolation areas	5	4	5	5	4	4	NR	4	NR	5	4	5	5	4	4.6	4.4

Use of PPE for health workers	5	5	5	5	4	4	NR	4	NR	4	4	5	4	4	4.4	4.4

Use of antiviral drugs for treatment	5	5	5	4	4	4	NR	4	NR	5	4	5	4	4	4.4	4.4

Use of antiviral drugs for prophylaxis	5	5	5	3	4	4	NR	3	3	4	3	5	4	4	4.0	4.0

Focus on hospital infection control	4	4	5	4	5	4	NR	3	NR	5	3	5	5	4	4.4	4.1

More preparation for medical surge	4	4	4	3	4	4	NR	3	NR	5	3	5	5	4	4.0	4.0

**MEAN**	4.6	4.5	4.9	4.0	4.1	4.0	--	3.6	3.0	4.8	3.5	4.9	4.4	4.0	**4.3**	**4.2**

***Disease Prevention and Control - Communities***																

Containment measures (from WHO)	4	4	4	4	4	4	NI	4	NR	5	3	4	5	4	4.0	4.1

Border control measures	4	4	NI	4	4	4	NR	3	NR	5	3	5	4	4	3.8	4.1

Restrictions of travel	NR	NI	NI	3	NI	3	NI	2	NI	NI	NI	NI	4	4	4.0	3.0

Restrictions of public gatherings	3	NI	NI	4	NI	3	NI	2	NI	5	NI	2	4	4	3.5	3.3

Closure of any schools	4	NI	NI	4	NI	4	NI	NI	NR	5	3	3	3	4	3.3	4.0

**MEAN**	3.8	4.0	4.0	4.0	4.0	3.6	--	2.8	--	5.0	3.0	3.5	4.0	4.0	**3.7**	**3.7**

***Risk Communications***																

Spokesperson(s) identified	5	5	5	4	4	4	NR	4	5	4	4	4	5	4	4.7	4.1

Public risk communications/targeted	5	4	5	3	4	4	NR	3	3	5	4	4	4	4	4.2	3.9

Public risk communications/broad	4	4	4	4	3	3	NR	3	4	5	3	5	4	4	3.8	4.0

Message consistency for public	4	5	5	4	4	4	NR	3	5	5	4	4	4	4	4.3	4.1

Messages aimed to avoid public panic	NR	4	3	4	4	4	NR	4	5	5	3	3	5	4	4.0	4.0

Communications w/MBDS country	NR	5	NI	3	NI	3	NR	4	3	5	3	NI	4	4	3.3	4.0

Communications w/MBDS office	5	5	NI	4	NI	3	NR	4	4	4	NI	NI	4	4	4.3	4.0

Communications w/external partner	5	5	NI	3	NI	1	NR	4	4	3	NI	NI	4	4	4.3	3.3

**MEAN**	4.7	4.6	4.4	3.6	4.0	3.3	--	3.6	4.1	4.5	3.5	4.0	4.3	4.0	**4.1**	**3.9**

1. CAMB = Cambodia; CYUN = China Yunnan-Province; CGUA = China Guangxi Province; LAOS = Laos; MYAN = Myanmar; THAI = Thailand; VIET = Vietnam

2. Ratings of activity performance measured on a Likert Scale where 1 = very poor, 2 = poor, 3 = average, 4 = good and 5 = excellent

3. NI = not implemented; NR = no response

Cambodia's ratings of its perceived performance overall were higher than those for other countries, especially for surveillance and information sharing (4.9 in 2009 and 4.5 in 2010 for Cambodia compared to 4.1 and 4.1, respectively, overall), disease prevention and control in health facilities (4.6 and 4.5 compared to 4.3 and 4.2, respectively), and risk communications (4.7 and 4.6 compared to 4.1 and 3.9, respectively). By 2010, Myanmar perceived its performance related to disease prevention and control activities in communities to be excellent (5.0 overall mean), higher than all other countries. Half to two-thirds of countries perceived the quality of their response efforts for surveillance, facility-based interventions and risk communications as lower overall post-pandemic, compared to their initial 2009 assessments. In contrast, only one country rated its overall performance for community-based interventions as lower post-pandemic (2010) compared to 2009. Myanmar and Thailand felt their performance had improved, in all areas reported, by the end of the pandemic; Vietnam also felt its performance in surveillance and information sharing had improved, and Cambodia also considered its community-based interventions as better post-pandemic than in the early stage of the pandemic.

### How did countries perceive their actual response performance in comparison to hypothetical performance of the same activities in 2006?

Table 4 presents comparisons of subjective ratings of actual performance in 2009 and 2010 to hypothetical performance of each activity had it been undertaken in 2006. For surveillance and information sharing, Cambodia and Thailand felt that their performance in 2009 was better in all areas compared to how they may have performed if they were to have responded to a pandemic in 2006 (mean difference of at least 1); by 2010, China-Guangxi and Laos also perceived differences of this magnitude in comparison with their hypothetical performance in 2006. China-Yunnan identified an area for improvement in the future: establishment of hotlines for reporting possible cases (performance judged worse in 2009 than it might have been in 2006). In the area of disease prevention and control in health facilities, Cambodia and Thailand considered their 2009 performance substantially better than it would have been in 2006; by 2010, Myanmar also considered its performance better than it would have been in 2006. Both Yunnan and Guangxi provinces of China and Vietnam felt that their good to excellent performance in 2009 was no different than it would have been in 2006. With regard to disease prevention and control in the community, again, Cambodia and Thailand considered their 2009 performance at least one point better on average compared to 2006; and again, by 2010 Myanmar felt its performance had improved to this degree as well. Finally, with regard to risk communications, Cambodia and Thailand again initially considered their performance to have been at least one point better on average than it would have been in 2006, but by 2010, only Cambodia and China-Guangxi perceived their performance for risk communications as at least one point better than in 2006.

**Table 4:** Subjective comparisons of actual (2009, 2010) to hypothetical (2006) response performance^1, 2^

ACTIVITY^2^	CAMB		CYUN		CGUA		LAOS		MYAN		THAI		VIET		MEAN	
	
	'09	'10	'09	'10	'09	'10	'09	'10	'09	'10	'09	'10	'09	'10	'09	'10
***Surveillance and Information Sharing***																

Development/use of case definition	1	0	0	-1	1	2	NR	3	NR	1	2	3	1	0	1.0	1.1

Active case finding/hospitals & clinics	3	0	1	0	2	2	NR	3	NI	1	1	0	1	0	1.6	0.9

Active case finding/communities	3	0	0	0	2	2	NR	3	NR	1	1	0	0	0	1.2	0.9

Active case finding/ports of entry	4	2	1	1	1	2	NR	NI	NR	1	2	2	0	0	1.6	1.3

Electronic reporting of cases	1	1	0	0	1	2	NI	0	NR	1	2	NR	0	0	0.8	0.7

Hotline for reporting possible cases	1	2	-1	0	0	2	NR	3	NR	1	2	2	1	0	0.6	1.4

Initial testing in district/provincial lab	NR	3	0	-1	1	2	NI	NI	NI	1	1	NI	0	0	0.5	1.0

Any other active surveillance activities	3	3	0	-2	0	2	NR	1	NR	1	2	NR	1	0	1.2	0.8

Rapid Response Team mobilization	2	1	0	-1	0	3	NR	0	NR	0	1	3	0	0	0.6	0.9

Communication w/cross-border site(s)	1	1	0	-1	NI	3	NR	0	NR	1	1	NI	0	0	0.5	0.7

Sharing info w/MBDS country	1	2	NI	-1	NI	3	NR	0	NR	0	1	NI	0	0	0.7	0.7

Sharing info w/MBDS Coordinator	1	1	1	-1	NI	3	NR	0	NR	0	1	NI	0	0	0.8	0.5

**MEAN**	1.9	1.3	0.2	-0.6	0.9	2.3	--	1.3	--	0.8	1.4	1.7	0.3	0	**0.9**	**0.9**

***Disease Prevention and Control - Health Care Facilities***																

Communications w/health workers	2	1	0	-1	0	1	NR	2	NR	1	1	1	0	0	0.6	0.7

Use hospital admission criteria	1	2	0	-1	NI	1	NR	1	NR	1	2	1	0	0	0.8	0.7

Preparation or use of isolation areas	1	2	0	0	0	0	NR	2	NR	2	2	1	0	0	0.6	1.0

Use of PPE for health workers	2	1	0	0	0	0	NR	0	NR	1	-1	1	0	0	0.2	0.4

Use of antiviral drugs for treatment	2	2	0	-1	0	0	NR	1	NR	2	2	1	0	0	0.8	0.7

Use of antiviral drugs for prophylaxis	1	2	0	-1	0	1	NR	0	-2	1	1	1	0	0	0.0	0.6

Focus on hospital infection control	2	1	0	-1	0	0	NR	1	NR	2	1	0	0	0	0.6	0.4

More preparation for medical surge	2	1	0	-1	0	1	NR	1	NR	2	1	0	0	0	0.6	0.6

**MEAN**	1.6	1.5	0	-0.8	0	0.5	--	1.0	-2.0	1.5	1.1	0.8	0	0	**0.5**	**0.6**

***Disease Prevention and Control - Communities***																

Containment measures (from WHO)	1	3	NR	-1	0	0	NI	2	NR	2	1	3	0	0	0.5	1.3

Border control measures	2	3	NI	-1	0	0	NR	1	NR	2	2	1	0	0	1.0	0.9

Restrictions of travel	NR	NI	NI	-1	NI	-1	NI	0	NI	NI	NI	NI	0	0	0.0	-0.5

Restrictions of public gatherings	2	NI	NI	-1	NI	-1	NI	0	NI	2	NI	1	0	0	1.0	0.2

Closure of any schools	1	NI	NI	-1	NI	0	NI	NI	NR	2	2	1	0	0	1.0	0.4

**MEAN**	1.5	3.0	--	-1.0	0	-0.4	--	0.8	--	2.0	1.7	1.5	0	0	**0.7**	**0.5**

***Risk Communications***																

Spokesperson(s) identified	1	2	0	-1	0	1	NR	0	0	1	2	1	0	0	0.5	0.6

Public risk communications/targeted	1	1	0	-1	0	1	NR	2	-1	1	2	0	0	0	0.3	0.6

Public risk communications/broad	1	1	1	-1	0	0	NR	2	0	1	2	0	0	0	0.8	0.4

Message consistency for public	1	2	0	-1	0	0	NR	1	NR	1	2	0	NR	0	0.8	0.4

Messages aimed to avoid public panic	NR	1	0	-1	0	0	NR	2	NR	1	1	2	0	0	0.3	0.7

Communications w/MBDS country	NR	1	NI	-1	NI	2	NR	0	NR	0	1	NI	0	0	0.5	0.3

Communications w/MBDS office	1	1	NI	-1	NI	2	NR	0	-1	0	NI	NI	0	0	0.0	0.3

Communications w/external partner	1	1	NI	-1	NI	2	NR	0	-1	0	NI	NI	0	0	0.0	0.3

**MEAN**	1.0	1.3	0.2	-1.0	0.0	1.0	--	0.9	-0.6	0.6	1.7	0.6	0	0	**0.4**	**0.5**

1. CAMB = Cambodia; CYUN = China Yunnan-Province; CGUA = China Guangxi Province; LAOS = Laos; MYAN = Myanmar; THAI = Thailand; VIET = Vietnam

2. NI = not implemented; NR = no response

### What factors contributed to successful responses to 2009-H1N1 in each country?

Table 5 presents subjective ratings of the contribution of different planning-related factors, including specific aspects of MBDS cooperation, to the success of each country's pandemic response performance. MBDS Country Coordinators considered pandemic preparedness plans at national, provincial and/or local levels to have been the most important factor contributing to the success of their pandemic responses, with average scores of 4.7 and 4.6 in 2009 and 2010, respectively. The second most important factor was strong political leadership (average scores of 4.4 in both 2009 and 2010). The three factors tied as third most important post-pandemic were the government's structure/management, the involvement of ministries in addition to the Ministry of Health, and having carried out pandemic preparedness exercises - each scored 4.3 on average in the 2010 survey. The 2010 post-pandemic survey indicated that international cooperation, including the 6-country MBDS agreement (mean score 3.4) and action plan (3.7), communications with other MBDS countries (3.6) and with their external partners (3.4), and country obligations under the revised WHO International Health Regulations (3.7) were viewed as less important contributors to successful country responses, in reflecting on overall responses after the pandemic had ended. Thailand rated MBDS cooperation as essentially irrelevant to its responses (scores of 1 for each such item, on a scale of 1-5).

**Table 5:** Subjective ratings of factors that contributed to success in pandemic response^1, 2, 3^

ACTIVITY	CAMB		CYUN		CGUA		LAOS		MYAN		THAI		VIET		MEAN	
	
	'09	'10	'09	'10	'09	'10	'09	'10	'09	'10	'09	'10	'09	'10	'09	'10
National, provincial and/or local pandemic preparedness plan	5	5	5	4	4	4	5	5	5	5	4	5	5	4	4.7	4.6

National (provincial) political leadership	5	5	5	4	4	5	5	4	5	5	2	4	5	4	4.4	4.4

Preparedness structure/management in government	5	5	5	4	3	4	5	5	5	5	3	3	5	4	4.4	4.3

Ministries in addition to MOH	4	5	4	4	4	3	5	5	5	5	4	4	5	4	4.4	4.3

MBDS MOU	5	5	4	3	2	3	4	4	5	4	2	1	5	4	3.9	3.4

MBDS action plan	4	5	4	4	3	4	4	4	4	4	2	1	5	4	3.7	3.7

MBDS - other plan	4	4	4	3	3	3	3	3	3	4	NA	1	5	4	3.7	3.1

MBDS country partner(s)	4	5	4	3	3	3	3	5	3	4	NA	1	5	4	3.7	3.6

MBDS development partner(s)	4	5	4	3	3	3	3	5	3	3	NA	1	5	4	3.7	3.4

TTX or other exercises (national, provincial and/or local)	4	5	5	4	3	4	5	4	3	4	3	5	5	4	4.0	4.3

International Health Regulations	4	5	5	3	3	4	5	2	3	4	3	4	4	4	3.9	3.7

1. CAMB = Cambodia; CYUN = China Yunnan-Province; CGUA = China Guangxi Province; LAOS = Laos; MYAN = Myanmar; THAI = Thailand; VIET = Vietnam.

2. MOH = Ministry of Health; MOU = Memorandum of Understanding

3. NA = not applicable

### Qualitative assessment of strengths and areas for improvement

With regard to perceived strengths in the pandemic responses of MBDS countries, two themes stood out based on our qualitative analysis of the comments offered by the survey participants. The first theme relates to surveillance and information sharing within countries, and the second theme relates to surveillance and information sharing across countries. Within countries, participants noted the ability of their country level surveillance systems to exchange information efficiently. These systems were referred to as: good (Cambodia), reliable (China-Yunnan), critical (Myanmar), and the most successful aspect of their country's response (Vietnam). Across countries, participants noted the importance of MBDS to enable timely coordinated regional response (Cambodia), detect disease at cross border sites (Myanmar), and prevent the spread of the virus (China-Guangxi).

Two key themes also emerged from comments about areas of response that MBDS countries perceived as still needing improvement. The first theme relates to the desire to continue to improve communications within countries, and the second theme relates to the desire to continue to improve communications across MBDS countries. Within countries, participants identified risk communication as a major challenge. Some noted that public health workers were "not fully trained to communicate with panicky citizens" or that more "health education" for the public was necessary. Participants also noted that MBDS communications across countries needed to be improved and sustained (Cambodia), expanded and strengthened (China-Guangxi), and that more communications in general were needed (Vietnam). One country noted that existing communications were the "bare minimum of regional collaboration" (Cambodia).

Participants also commented on plans to improve their response in the future. They identified plans for improvement for each of the three broad areas discussed. For example, plans to improve surveillance and information sharing included better electronic reporting and active case finding, sustained availability of hotline support, joint outbreak investigation across MBDS borders and surveillance reaching out to the community level. These health leaders hoped to improve influenza prevention and control through building better capacity for laboratory diagnosis; ensuring better access to vaccines, drugs and protective equipment; building better local pandemic response capacity and better surge capacity. Needed improvements for communications among responders and with the public include development and use of better information and communications technologies, building capacity for public risk communications and judicious use of such communications. These leaders also identified ways to strengthen the collaboration among the MBDS countries.

## Discussion

This paper presents an analysis of the subjective assessment of performance at two points in time related to MBDS countries' responses to the 2009-H1N1 pandemic. Performance early during the pandemic and post-pandemic was reported by senior health leaders in six countries in the Mekong Basin, all of whom were heavily involved in their countries' pandemic response. These countries had formally organized themselves eight years earlier for a new type of transnational cooperation - a sub-regional network for cooperation in disease surveillance, prevention and control that reached from the central government level out to provincial and local cross-border operational sites. Based on the judgments of MBDS Country Coordinators, elements of pandemic preparedness at national and sub-regional levels identified as problematic from the 2006-2007 MBDS exercises (many of the specific items included in the surveys reported here) were performed relatively early and well in the responses to 2009-H1N1 influenza, as perceived both early during the pandemic (June 2009) and post-pandemic (December 2010). Important contributors to these successes included extensive pandemic influenza planning, political leadership and government structure, the involvement of multiple government ministries in the pandemic response, and having carried out pandemic preparedness exercises including the MBDS-sponsored exercises in 2006-2007, which were the first exercises ever carried out in all MBDS countries except Thailand. Cambodia and Thailand in particular, and Myanmar to only a slightly lesser degree, felt that their public health responses were better in 2009 and 2010 than they would have been in 2006.

Most performance indicators related to responses within countries; the role of MBDS cooperation in contributing to better pandemic preparedness in these countries cannot be assessed directly from our surveys. However, while the quantitative scores indicate that MBDS cooperation was not the principal driver of pandemic response in the individual countries (a reasonable and legitimate perspective), performance in surveillance and information sharing and risk communications with other MBDS countries and with the MBDS coordinator were generally judged as good. Moreover, the qualitative responses also suggest that most MBDS countries value MBDS communications and cooperation and seek to improve them - Cambodia, China, Myanmar and Vietnam all commented explicitly in this regard. These findings lend support to a general conclusion that MBDS cooperation added value to the pandemic responses across these countries. Somewhat troubling, however, is the wide variability (across the entire 5-point range of ratings, Table 5) in the value different countries attached to MBDS cooperation, or at least to the specific elements included in the surveys--the MOU, action plan, and role of other MBDS countries.

The ultimate outcomes of interest are better system capabilities and better health in the populations of these countries. Findings from the surveys reported here suggest that the MBDS cooperation added value to their public health preparedness and pandemic response. Earlier experiences had already included several instances of cooperative cross-border outbreak detection and response. The quantitative and qualitative responses in the surveys reported here pointed to a few areas for further attention, including laboratory capacity, risk communications, electronic communications and local cross-border cooperation. Several of these are already included in the MBDS Master Plan for 2011-2016, the core strategies of which capture some of the key capabilities needed for pandemic preparedness: cross-border cooperation in surveillance and response; coordination between animal and human health; community-based surveillance; epidemiology capability; information and communications capability; laboratory capability; risk communications; and policy research [28]. It is reasonable to expect that these capabilities, within the MBDS community and across this and other regions, will improve both public health systems and health outcomes by enabling more-timely and better detection, communications, coordinated containment, and control of the next small outbreak or the next major pandemic.

The strengths of this paper include its focus on sub-regional response (an emerging trend in global public health), the use of surveys both during and after the pandemic (to assess early progress and needs for improvement as well as overall performance after the hectic pace of pandemic response had subsided) and the fact that the country surveys, though not large in number, were completed by senior health officials who were knowledgeable and heavily involved in their country's pandemic response. The weaknesses of the paper can be broken into three categories: the reliance on subjective responses from a small number of respondents, the inability to assess how different activities impacted outcomes across countries, and the limited ability to assess how and the extent to which the MBDS sub-regional collaboration influenced their pandemic response.

The surveys reflect subjective judgments of individual health leaders in the six MBDS countries related to their respective country's pandemic response early during the pandemic and again post-pandemic. While more objective measures, more rigorous data collection methods, and the views from a larger number of country officials may have enhanced the validity of the results, we believe that these results had sufficient face validity for the practical purposes intended - to guide further improvements during the pandemic (from the 2009 survey) and provide useful insights to guide the future actions (from the 2010 survey).

An important aspect of quality improvement is the ability to iteratively test different interventions to see how effective each one is and which interventions work better than others. In this work we were not able to directly assess whether or the extent to which specific activities taken by each country resulted in different outcomes. For example, we cannot directly assess whether or not the decision of Cambodia to limit travel resulted in better outcomes (such as slower disease spread) than the other five countries that did not restrict travel. We also cannot link perceived performance quality to actual outcomes.

Our survey contained some information on how MBDS countries collaborated together during the pandemic. Our ability to assess what aspects of that collaboration resulted in optimal responses is limited. In addition, we have only a limited knowledge of how MBDS collaborative planning prior to the pandemic directly or indirectly impacted their national or collective sub-regional response. In a more ideal circumstance, a baseline survey would have been conducted prior to all MBDS planning and would not require participants to hypothetically conjecture how their responses might have differed prior to their focused pandemic planning efforts.

MBDS was founded upon a principle of coordinated actions for the common regional good and rapid and open communications across the MBDS community. Despite this, there was considerable variability in the timing of initiation of communications across MBDS countries and with the MBDS Coordinating Office during the 2009-2010 response. From their early experiences, Country Coordinators identified some priorities for MBDS action during 2009-2010, and they offered further ideas for improvement post-pandemic. By the end of the pandemic, some countries felt their performance in one or more broad areas and for specific activities had improved since 2009, but other countries rated their 2010 performance as less effective than that in 2009.

We believe it was useful to examine public health system performance in a real situation--the 2009-H1N1 pandemic. As might be expected, the MBDS leaders identified areas where early pandemic response was judged to be good to excellent and other areas where they intended to target their improvement efforts in the near term. The 2009 survey results were presented to a large MBDS conference in August 2009 in Kunming, China, and MBDS leaders incorporated some of the survey's findings into their MBDS operational plans for the next two years. Most of these plans have not yet received funding support, so assessment of actual improvements since 2009 has been limited. The information from the follow-up survey should also feed into the planning process within countries and across the MBDS cooperative community.

## Conclusions

The public health emergency response capacity that MBDS countries had built through training and simulation exercises, national and sub-regional planning, multi-sector engagement, and political leadership in each country enabled what health leaders across the MBDS community perceived as effective responses in areas that had been identified as problematic during the 2006-2007 MBDS tabletop exercises. Health leaders from most but not all countries perceived that MBDS cooperation helped drive and thus added value to their responses to the 2009-H1N1 pandemic. Surveillance capacity within countries and surveillance information sharing across countries, longstanding focus areas of MBDS programming, were cited as strengths during the pandemic response. Several specific areas cited as needing further improvement are already core strategies in the MBDS Action Plan for 2011-2016. However, the familiarity and mutual trust that MBDS countries have built over the decade of cooperation did not carry over in a consistent way in terms of the importance of MBDS network cooperation in each country's pandemic response. It may be worthwhile to explore the reasons for this, as yet another avenue for enhancing future cooperation and future collective response to cross-border and trans-national disease threats.

Self-organized sub-regional consortia for cooperation in disease surveillance are increasingly recognized as a new and important element in global disease prevention and control, which has become very cross-border and trans-national in nature [24]. MBDS is one of the longest-standing of such sub-regional cooperative groups and has been described as an "innovative cross-border initiative." [17] The true value of sub-regional surveillance networks like MBDS remains unclear. Countries involved in these networks must determine the appropriate role for their network vis-à-vis national efforts and how to sustain collaboration across their countries. Our findings suggest that more research is needed to understand the characteristics of networking that make the most sense and that will result in the best shared outcomes.

## Competing interests

The authors declare that they have no competing interests.

## Authors' contributions

MM and DJD designed and conducted the study. MM led the writing and analysis. DJD contributed substantially to the analysis and writing. Both authors have read and approved the final manuscript.
